# Lipid Lowering Drugs in Acute Coronary Syndromes (ACS)

**DOI:** 10.1007/s11883-023-01163-6

**Published:** 2023-11-28

**Authors:** Natalie Arnold, Wolfgang Koenig

**Affiliations:** 1 Department of Cardiology, University Heart & Vascular Center Hamburg, University Medical Center Hamburg-Eppendorf, Hamburg, Hamburg Germany; 2 German Center for Cardiovascular Research (DZHK), Partner Site Hamburg/Kiel/Luebeck, Hamburg, Germany; 3 Deutsches Herzzentrum München, Technische Universität München, Lazarettstr. 36, 80636 Munich, Germany; 4 German Centre for Cardiovascular Research (DZHK), Partner Site Munich Heart Alliance, Munich, Germany; 5 Institute of Epidemiology and Medical Biometry, University of Ulm, Ulm, Germany

**Keywords:** Lipid-lowering medication, Acute coronary syndrome, Dyslipidemia, Atherosclerotic cardiovascular disease, PCSK9 inhibitors

## Abstract

**Purpose of Review:**

The purpose of this review is to critically discuss whether more aggressive lipid-lowering strategies are needed in patients with acute coronary syndromes (ACS).

**Recent Findings:**

Currently, available data on early (in-hospital/discharge) administration of potent lipid-lowering drugs, such as proprotein convertase subtilisin/kexin 9 (PCSK9) inhibitors in patients during the vulnerable post-ACS phase, have clearly demonstrated clinical efficacy of the “strike early and strike strong” approach not only for rapid reduction of low-density lipoprotein cholesterol (LDL-C) to unprecedentedly low levels, but also for associated favorable composition of coronary plaque.

**Summary:**

Intensive lipid-lowering therapy with rapid achievement of the LDL-C treatment goal in ACS patients seems reasonable. However, whether such profound LDL-C reduction would result in additional benefit on the reduction of future CV events still has to be established. Thus, data addressing CV outcomes in such vulnerable patients at extreme CV risk are urgently needed.

## Introduction

The most vulnerable phase during the whole course of the atherosclerotic cardiovascular disease (ASCVD) process is the early phase of an acute coronary syndrome (ACS), which is associated with a significant case-fatality and a high rate of recurrent cardiovascular (CV) events [1, 2]. Indeed, data from Swedish national registries, including 108,315 individuals demonstrated that 10.2% of patients, admitted to the hospital for myocardial infarction (MI), died within the first 7 days after the index event with an additional mortality of 12–13% during the next 12–24 months in initial survivors [1]. Moreover, one in five patients discharged with MI had a subsequent CV event (stroke, MI, or CV death) in the first year after the index MI [1]. Thus, there is a clear need of aggressive preventive measures in such patients with an “extreme” CV risk to improve outcome. Interestingly, the exact mechanisms, which might be responsible for plaque instability, the most broadly accepted underlying pathology of ACS, are still not entirely understood, since the pathophysiology of ACS on a molecular level is much more complex than previously assumed [3, 4]. A tight interplay between lipids, inflammation, and platelets seems to be the most important in this regard [4]. Recently, an emerging role of proprotein convertase subtilisin/kexin 9 (PCSK9) as a central player of such dangerous trias has been proposed with regard to not only lipoprotein disturbances but also increased platelet reactivity, thrombus formation, MI expansion, and finally inflammation [5].

During recent years, aggressive treatment of dyslipidemias besides acute coronary intervention with subsequent individualized antiplatelet strategies became the central strategy in this high-risk situation. The latest European Society of Cardiology/European Atherosclerosis Society (ESC/EAS) 2019 guidelines for dyslipidemia management [6••], however, do not distinguish between acute and chronic coronary syndrome, proposing “one size fits all” lipid-lowering treatment algorithm, despite strong differences in patient’s baseline risk. More importantly, they introduced rather a two-step approach to low-density lipoprotein cholesterol (LDL-C) goals, starting with high intensity statins early after admission (within the first 1–4 days of hospitalization) (Class IA recommendation) with further addition of a non-statin lipid-lowering drug (e.g., ezetimibe or PCSK9 inhibitors (PCSK9i)) if the LDL-C target of < 55 mg/dl has not been achieved. Such approach, however, requires additional follow-up (FU) visits with a routine re-evaluation of LDL-C levels and in many cases uptitration of lipid-lowering therapy (LLT) and would rather result in an “artificial” prolongation in LDL-C goal attainment, which might be expected in the best case scenario only after 4 months post-MI. So, the important question was as follows: Would the guideline-recommended LDL-C lowering strategy in ACS patients be sufficient to reduce CV risk properly? Or, to be more precise, do we really do enough for our patients, who are at an extremely high CV risk?

To this end, recent data from the SWEDEHEART registry, including 40,607 patients with ACS, are of importance [7•]. Evaluating the relationship between statin intensity and magnitude of LDL-C reduction on subsequent CV risk after an index event, Schubert et al. impressively showed a strong benefit of early treatment with high-intensity statins (HIS) on the reduction of future risk. Those subjects, discharged on HIS and achieving the largest (75^th^ percentile) LDL-C reduction between the index event and 6–10 weeks later, demonstrated the highest benefit of early HIS with a markedly reduced incidence for major adverse CV events (MACE) or CV and even total mortality by ~ 20 to 30% compared to those with a smaller LDL-C reduction. In contrast, subjects with either a low response to LLT or even with increasing LDL-C were at highest risk for MACE over a 4-year FU period, as one might have expected. These findings not only reinforce the well-established benefits of LDL-C lowering on future CV events, but also underscore the clinical relevance of rapid reduction of LDL-C in the ACS setting. They also provide a further argument in favor of a recently proposed “strike early and strike strong” approach [8••], implying an efficient, pragmatic, and immediate LDL-C reduction in the early post-ACS phase.

The present review critically discusses the currently available lipid-lowering approaches in the early management of ACS patients.

## Statins, Ezetimibe, and Bempedoic Acid

For more than 20 years, we know that early and intensive LDL-C lowering results in considerable risk reduction in patients after ACS. Initial evidence came from the MIRACL (Myocardial Ischaemia Reduction with Aggressive Cholesterol Lowering) trial [9], addressing the early benefits of 80 mg of atorvastatin, initiated within 24 to 96 h after an index event in 3086 patients with ACS without ST-segment elevation. Compared to placebo, those on atorvastatin demonstrated a 16% reduction of recurrent ischemic events within the first 16 weeks, and the benefit of atorvastatin therapy (i.e., event curves separation) was already seen after the first 30 days of treatment. Furthermore, in 2004, Cannon et al. investigated 4162 ACS patients, participating in the PROVE-IT TIMI 22 trial (Pravastatin or Atorvastatin Evaluation and Infection Therapy), who were randomized 1:1 to receive 40 mg of pravastatin daily (standard therapy) versus 80 mg of atorvastatin daily (high intensive therapy) within 1 week after the acute event [10]. Approximately, a 16% lower risk for MACE in favor of atorvastatin could be demonstrated at 2 years. More importantly, an even stronger risk reduction of 28% (hazard ratio (HR) 0.72 (95% confidence interval (CI) 0.52–0.99); *p* = 0.046) was seen on high intensity atorvastatin therapy, if a composite end-point was determined at 30 days [11•]. Recently published observational data by Kim et al. [12•] investigated the timing of statin initiation in relation to long-term clinical outcomes and demonstrated that early statin initiation within 48 h after admission was associated with a significant reduction of MACE by approximately 20% during a FU of 4 years compared to the later start of statin therapy (≥ 48 h), thereby suggesting in-hospital statin-therapy initiation within the first 2 days after admission as an optimal timing to consider. Interestingly, they found no differences in the incidence of MACE between the early (< 24 h) and later statin initiation (24–48 h), although an initiation of statin treatment within the first 24 h significantly lowered in-hospital mortality rate.

With regard to the role of ezetimibe, a Niemann-Pick C1-like protein inhibitor, which, according to guidelines [6••], should be added to HIS after 4–6 weeks if LDL-C is still above the target value, we have only limited evidence on its role for CV risk reduction in the acute coronary setting. The first trial, demonstrating a reduction of adverse events with ezetimibe added to statins after ACS was IMPROVE-IT (Improved Reduction of Outcomes: Vytorin Efficacy International Trial), including 18,144 patients with ACS within the preceding 10 days, where 10 mg ezetimibe or placebo were added to simvastatin 40–80 mg daily [13]. A modest yet statistically significant risk reduction for a composite MACE was seen at 6 years in the simvastatin-ezetimibe group versus simvastatin-monotherapy with a HR of 0.93 (95% CI 0.89–0.99). In a recent open-label HIJ-PROPER study (Heart Institute of Japan-proper level of lipid lowering with pitavastatin and ezetimibe in acute coronary syndrome), including 1734 ACS patients, a combination of both pitavastatin plus ezetimibe produced greater CV benefits over 4 years, compared to statin monotherapy only in ACS patients with higher cholesterol absorption (HR 0.71 (95% CI 0.56–0.91) [14].

Although bempedoic acid (BA), an inhibitor of intracellular cholesterol biosynthesis, has been recently introduced in the clinical setting [15] and might represent another promising option for dyslipidemia treatment to reduce future CV risk, no data are available in ACS patients so far.

Most importantly, a triple combination of statins, ezetimibe, and BA theoretically has the potential to effectively reduce LDL-C by 75% from baseline. The question to be raised here is whether a LDL-C reduction, which might be achieved by such triple combination, would also result in a similar risk reduction of MACE as that seen during treatment with PCSK9i on top of HIS.

### PCSK9 Inhibition

The development of PCSK9 inhibitors has revolutionized the current treatment of dyslipidemia [16, 17]. It started 20 years ago with impressive genetic studies in individuals with either loss- or gain-of-function PCSK9 gene mutations and consecutive low or high CV disease risk, respectively [18–20]. To date, there are only two approved modalities to inhibit PCSK9 activity. Alirocumab and evolocumab are fully human monoclonal antibodies (mAbs) targeted against PCSK9, whereas inclisiran represents a first-in-class cholesterol-lowering small interfering ribonucleic acid (siRNA), targeting PCSK9 messenger RNA (mRNA) in hepatocytes. In contrast to anti-PCSK9 mAbs, inclisiran inactivates PCSK9 by inhibition of its hepatic synthesis [17].

The addition of such treatment modalities to the modern lipid-lowering armamentarium was strongly supported by the results of large CV outcome trials (CVOTs) for both evolocumab (FOURIER (further cardiovascular outcomes research with PCSK9 inhibition in subjects with elevated risk) [21]) and alirocumab (ODYSSEY OUTCOMES (evaluation of cardiovascular outcomes after an acute coronary syndrome during treatment with alirocumab) [22]), showing a 50–60% reduction in LDL-C concentration during therapy with a mAb (on top of maximally tolerated statin therapy) being associated with an approximately 15% reduction of future CV risk over a mean follow-up of 2.3 or 2.8 years, respectively. Importantly, a pre-specified analysis of the FOURIER trial, including 5,711 patients with recent MI (< 12 months prior to randomization with the median time from the qualifying MI of 4.8 months) showed that evolocumab reduced the relative risk of MACE in these patients by 19% (HR 0.81 (95% CI, 0.70–0.93) compared to placebo, with first treatment effects seen at approximately 6 months [23]. In contrast, patients with a previous MI > 12 months showed only 8% relative risk reduction (HR, 0.92 (95% CI, 0.84–1.01) with event curves separation after 12 months. More recently, Schwartz et al. [24•] investigated the effect of a short duration of markedly LDL-C lowering to < 15 mg/dl achieved with statin and alirocumab on CV outcome within the post hoc analysis from the ODYSSEY OUTCOMES trial. This analysis demonstrated that even a short period of approximately 6 months of very intensive LLT resulted in very low LDL-C levels which were associated with prolonged CV risk reduction and a lower risk of MACE than statin monotherapy throughout the observation period. In case of inclisiran, the first CV outcome trial (CVOT)—ORION-4 (NCT03705234)—is still ongoing and estimated to be completed in 2026 (based on the information from ClinicalTrial.gov). ORION-4, which has recruited patients only in the UK and the USA, will be followed by a second CVOT, VICTORION-2 PREVENT (NCT05030428), which is currently underway worldwide.

Importantly, the implementation of PCSK9-targeted therapy might be even more relevant in the acute coronary setting [25]. Indeed, recent mechanistic data suggested additional detrimental effect of PCSK9 on the CV system, which are not only related to a pivotal pathophysiological role of PCSK9 in lipid regulation by degradation of the LDL receptor (LDLR) in hepatocytes [26]. For instance, the early ACS phase is highlighted, among others, by increased levels of PCSK9 in the circulation, which correlates with enhanced platelet activation, vulnerability of coronary plaque, and elevated inflammatory markers [reviewed in 27–29]. Also, data from the PCSK9-REACT study, including patients with ACS undergoing angioplasty and treated with ticagrelor or prasugrel, have confirmed the association between increased PCSK9 plasma level and residual platelet activity, which was further translated to an increased MACE risk at a 1 year follow-up among patients with elevated PCSK9 plasma levels at baseline (HR 2.61 (95% CI 1.24–5.52; *p* = 0.01) [30]. So, currently, available scientific evidence might therefore potentially justify an earlier initiation of PSCK9 therapy in ACS, especially taking into account that even a single subcutaneous administration of evolocumab (e.g., 140 mg in healthy volunteers) might decrease unbound PCSK9 by 97% within 15 min after injection [31]. Moreover, administration of the PCSK9i evolocumab in patients with ACS decreased markers of platelet activation and endothelial dysfunction in the early post-infarction period [32].

Despite all promising data, the application of PCSK9i in the acute coronary setting is only at the very beginning, since no data, addressing CV outcomes of early PCSK9i initiation in ACS patients, are available so far. Current evidence is rather related to the effect of PCKS9 inhibition on lipid levels as well as on coronary plaque morphology.

To date, there are several trials which have assessed the effects of PCSK9i treatment on LDL-C reduction within the acute phase after an ACS. The first trial—the EVACS trial (evolocumab in acute coronary syndrome) [33]—included 57 patients with ACS (60% were on previous statin therapy), who were randomized to a single dose of evolocumab SQ 420 mg or placebo within 24 h after the index event. Significant LDL-C reduction in the evolocumab group could be demonstrated already 24 h after application, with a decrease from 91.5 ± 35 to 70.4 ± 27 mg/dl in the evolocumab arm versus 89.6 ± 41 to 86.0 ± 40 mg/dl in the placebo arm. At discharge (at days 4 to 7), about 65% of patients in the PCSK9i-group achieved guideline-recommended LDL-C levels < 55 mg/dl (LDL-C 31.9 ± 23 mg/dl at discharge), compared to the placebo group, where only 23.8% of participants achieved LDL-C levels < 55 mg/dL according to the ESC/EAS guidelines (LDL-C 75.0 ± 32 mg/dl at discharge). In the larger EVOPACS (evolocumab for early reduction of LDL cholesterol levels in patients with acute coronary syndromes) study [34], efficacy and safety of in-hospital (i.e., no later than 4 days after hospitalization for ACS) initiation of 420 mg evolocumab on top of 40 mg atorvastatin daily were investigated among 308 ACS patients. After two doses of evolocumab given 1 month apart, LDL-C was reduced by 77.1 ± 15.8% (from 139.6 to 30.5 mg/dl), whereas in the placebo group, at week 8, it was only lowered by 35.4 ± 26.6% from 132.3 to 79.7 mg/dl. This resulted in LDL-C goal achievement in 90% already 8 weeks after an ACS compared with 11% on standard treatment [34]. With regard to alirocumab, in one small study (VCU-AlirocRT) with 20 NSTEMI patients, alirocumab was administrated within 24 h of NSTEMI presentation and showed a significant LDL-C reduction by 64 mg/dl from baseline only 14 days after treatment initiation (LDL-C reduction from 91 mg/dl at baseline to 73 and 28 mg/dl at 72 h and 14 days, respectively) (*p* = 0.02) [35]. Other data on the in-hospital use of alirocumab in post-ACS patients came from the EPIC-STEMI trial (effects of acute, rapid lowering of low density lipoprotein cholesterol with alirocumab in patients with ST segment elevation myocardial infarction undergoing primary PCI) [36]. Here, 68 STEMI patients were randomized to alirocumab (150 mg biweekly) or placebo on top of HIS therapy (97% of the alirocumab group and 100% in the sham-control group). From baseline to week 6 (with a median of 45 days), LDL-C levels were reduced by 72.9% in alirocumab patients versus 48.1% in the placebo group with a mean between-group difference of – 22.3%. Finally, the effect of early administration of inclisiran in ∼ 380 patients with a recent ACS (within 5 weeks) on LDL-C reduction and achievement of lipid targets at 1 year FU compared with standard therapy is currently being investigated in the VICTORION-INCEPTION trial (NCT 04873934).

Even more important are effects of PCSK9 inhibition on plaque stabilization and regression, especially taking into account that atherosclerosis has been considered for decades as an irreversible condition We have now evidence that evolocumab and alirocumab treatment, both on top of statins, modifies coronary plaque composition in patients with ACS, leading not only to a significant thickening of the fibrous cap but also resulting in regression of atheroma volume, as has been recently shown within the HUYGENS (high-resolution assessment of coronary plaques in a global evolocumab randomized study) [37•] and the PACMAN-AMI (effects of the PCSK9 antibody alirocumab on coronary atherosclerosis in patients with acute myocardial infarction) [38•] studies.

Within the HUYGENS trial [37•], 161 patients were randomized to receive evolocumab 420 mg monthly or placebo for 52 weeks. As expected, evolocumab therapy was associated with significantly greater LDL-C reduction from 140.4 ± 34.0 to 28.1 ± 25.4 mg/dl, than placebo (142.1 ± 32.3 to 87.2 ± 36.5) with absolute changes of − 114.2 ± 41.7 vs. − 55.3 ± 47.1 mg/dl (*p* < 0.001), allowing 86.4% vs. 20.0% patients to achieve the treatment threshold of LDL-C < 55 mg/dl. Further, assessment of plaque composition by intravascular imaging using optical coherence tomography (OCT) showed that evolocumab treatment led to plaque stabilization by increasing fibrous cap thickness by 39.0 µm (95% CI: 20.5–71.0) vs. + 22.0 µm (95% CI: 8.0–36.0) in the placebo group (*p* = 0.015), which was linear to LDL-C reduction. In addition, a decrease in maximum lipid arc (− 57.5 vs. − 31.4; *p* = 0.04) and macrophage index (− 3.17 vs. − 1.45 mm; *p* = 0.04) throughout the arterial segment was observed. Those data were complemented by the observation of greater plaque regression on evolocumab in patients with evaluable IVUS imaging, showing a significant reduction in percent atheroma volume (PAV) with evolocumab compared with placebo (− 2.29% vs − 0.61%; *p* = 0.009).

Alirocumab demonstrated very similar effects on coronary plaque composition and regression. The PACMAN-AMI trial [38•] enrolled 300 patients undergoing PCI for acute MI, who were randomly allocated 1:1 to receive either alirocumab or placebo. Alirocumab 150 mg was administered biweekly for 52 weeks with the first dose within 24 h after PCI. At week 52, the mean LDL-C level was found to be 74.4 mg/dl in the placebo group and 23.6 mg/dl on alirocumab therapy with a baseline LDL-C of 152.8 mg/dl. Again, incremental coronary plaque regression as well as lipid core reduction and plaque stabilization with alirocumab were seen (PAV changes from baseline in the alirocumab group − 2.13% vs. − 0.92% on placebo, *p* < 0.001; mean change in maximum lipid core burden index within 4 mm, − 79.42 with alirocumab vs − 37.60 on placebo, *p* = 0 0.006; mean change in minimal FCT 62.67 μm with alirocumab vs 33.19 μm with placebo, *p* = 0.001).

**Fig. 1:**
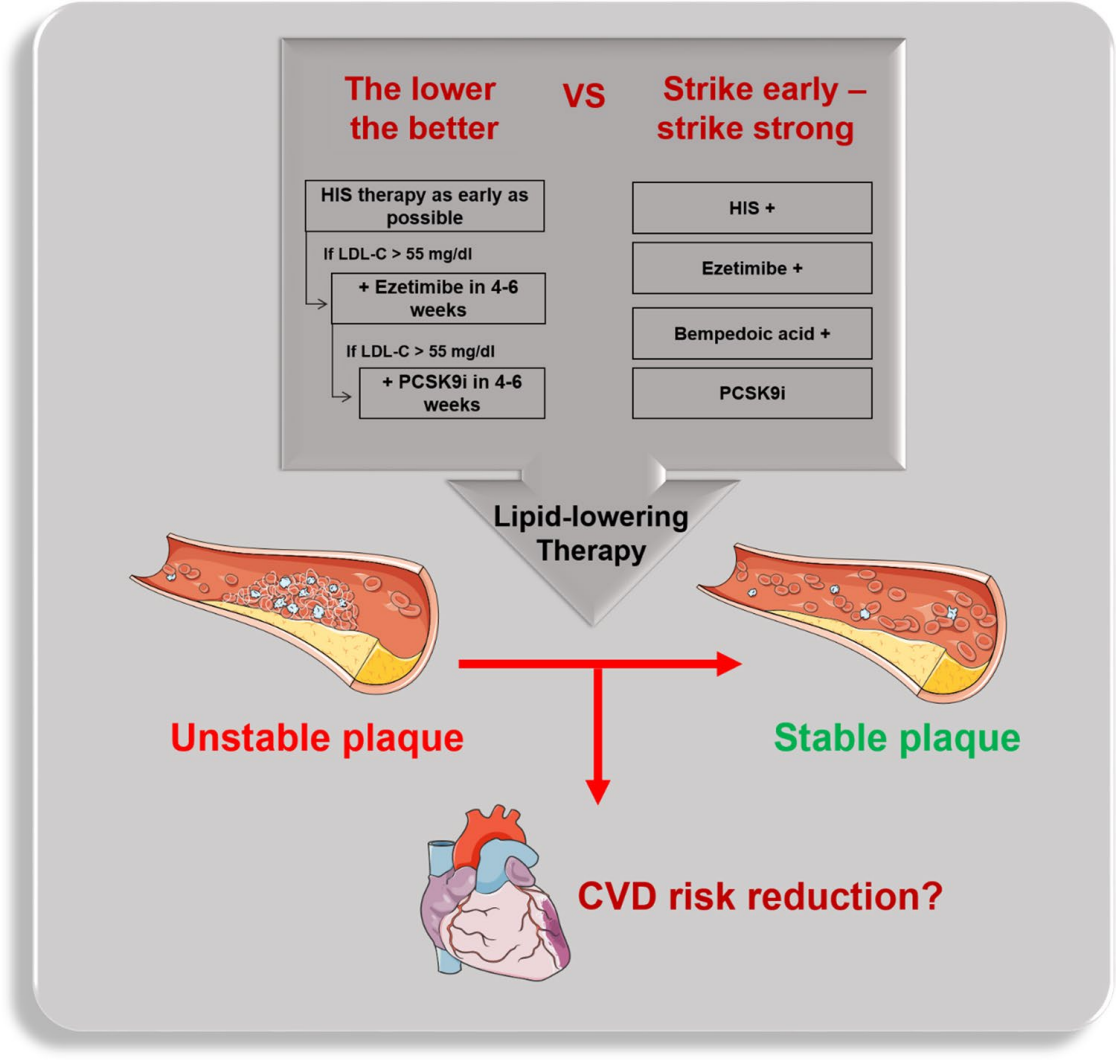
Paradigm shift for lipid lowering therapy in acute coronary syndrome and cardiovascular risk reduction. HIS, high intensity statins; PCSK9i, proprotein convertase subtilisin/kexin 9 (PCSK9) inhibitors; CVD, cardiovascular disease (source: the icons in this image are reproduced from Servier Medical Art by Servier. Reproduced from Servier Medical Art by Servier under a Creative Commons [CC BY 4.0] license)

Despite the fact that initiation of PCSK9i in-hospital not only decreases LDL-C to unprecedented low levels rapidly, but also might modify coronary plaque morphology favorably, the clinical benefits of early and aggressive lipid lowering leading to improved clinical outcomes is still uncertain (Fig. 1). Although the above mentioned studies have clearly demonstrated the feasibility and efficacy of such approach, also on a cellular level (plaque composition), they all were not powered for clinical outcomes [39]. So, there is an unmet need of future CVOTs to demonstrate the efficacy and safety of in-hospital PCSK9 inhibition on LDL-C lowering in ACS patients. Indeed, several CVOTs such as e.g. the EVOLVE-MI trial ((evolocumab very early after myocardial infarction) in 4000 ACS patients, randomized to evolocumab on top of routine lipid management versus routine lipid management alone irrespective of baseline LDL-C level (NCT05284747)) or the AMUNDSEN trial ((evolocumab or normal strategies to reach LDL objectives in acute myocardial infarction upbound to PCI) in 1666 ACS patients, randomized to a 12-month treatment with evolocumab 140 mg s.c. biweekly, with the first dose given prior to PCI (NCT04951856)) are already ongoing and are eagerly awaited.

## Conclusion

The management of global risk in extremely vulnerable patients with ACS remains challenging, given the complexity of treated patients. Insufficient LDL-C lowering still represents one of the most important issues in secondary CV prevention, leaving such vulnerable patients at unacceptable high CV risk. The combination of high-intensity statin and ezetimibe, if appropriately applied, might lead to an attainment of the proposed LDL-C target in 80% of the patients already at 4–6 weeks after ACS [40, 41], and further escalation with either bempedoic acid or PCSK9i would allow to reach ESC/EAS-LDL-C targets in all patients. However, what is still underappreciated so far is how fast we are indeed able to lower LDL-C successfully. We have encouraging evidence from mechanistic studies that early attainment of LDL-C goals results in favorable effects on plaque composition in such extremely high CV risk patients favoring a stable phenotype. First attempts to translate these findings into clinical practice have been already undertaken [8••, 42], suggesting early and intensive LDL-C lowering in the post ACS phase, the so-called strike early and strike strong.

Thus, it is undoubtful that the application of a PCSK9i in the early post-ACS phase on top to HIS/ezetimibe therapy leads to more effective and rapid LDL-C lowering, hereby insuring LDL-C target goal achievement in most patients still within the early and vulnerable phase. Nonetheless, at present, there is no clinical trial evidence that initiation of PCSK9i at the time of hospitalization translates into improved outcome. Thus, there is a clear need for such trials to confirm the validity of the “strike early and strike strong” approach.
